# Abnormalities of Otoacoustic Emissions in Myasthenia Gravis: Association With Serological and Electrophysiological Features

**DOI:** 10.3389/fneur.2018.01124

**Published:** 2018-12-20

**Authors:** Jongsuk Choi, Nam-Hee Kim, Soo-Hyun Park, Chang Gun Cho, Hyo-Jeong Lee, Sung Un Kim, Kyung Seok Park

**Affiliations:** ^1^Department of Neurology, Seoul National University Bundang Hospital, Seoul National University College of Medicine, Seoul, South Korea; ^2^Department of Neurology, Dongguk University Ilsan Hospital, Ilsan, South Korea; ^3^Department of Critical Care Neurology, Seoul National University Hospital, Seoul, South Korea; ^4^Department of Otorhinolaryngology, Dongguk University Ilsan Hospital, Ilsan, South Korea; ^5^Department of Otorhinolaryngology-Head and Neck Surgery, Hallym University College of Medicine, Chuncheon, South Korea

**Keywords:** myasthenia gravis, otoacoustic emissions, outer hair cells, cholinergic transmission, nicotinic acetylcholine receptors

## Abstract

**Objective:** To investigate whether otoacoustic emissions (OAEs) are impaired in patients with myasthenia gravis (MG) and whether such dysfunction is associated with serological and electrophysiological features of MG.

**Methods:** We tested 15 patients with MG (30 ears) and 10 healthy age- and sex-matched subjects (20 ears) for transiently evoked OAE (TEOAE) and distortion product OAE (DPOAE).

**Results:** Compared with controls, MG patients revealed a significant reduction in the amplitude of TEOAEs (*p* < 0.05) and DPOAEs at higher frequencies between 2,026 and 4,053 Hz (*p* < 0.05). In the subgroup analysis, TEOAE and DPOAE amplitudes were significantly lower in the acetylcholine receptor (AChR) antibody-positive group (*p* < 0.05) as well as in the repetitive nerve stimulation (RNS)-positive (*p* < 0.05) group. In particular, the OAE alteration significantly correlated with anti-AChR antibody titers. No significant difference of the OAEs was found between thymomatous and non-thymomatous MG or between purely ocular and generalized MG.

**Conclusions:** Our study confirms that OAEs reveal subclinical dysfunction of the cholinergic neurotransmission of cochlear outer hair cells and correlate well with electrophysiological and serological characteristics of MG patients. Our findings imply that the measurement of OAEs might increase the diagnostic accuracy and help to monitor the severity of MG.

## Introduction

Myasthenia gravis (MG) causes fatigable muscle weakness and is the most common autoimmune disorder affecting neuromuscular transmission (1). MG is diagnosed according to clinical symptoms and confirmed by one or more pharmacological, electrophysiological, or serological examinations (2); nonetheless, such diagnostic techniques are often unsuccessful, featuring an especially low sensitivity in cases of isolated ocular or bulbar MG (1, 2).

Although antibody targets characteristic of MG reportedly include muscle-specific kinase (MuSK) and lipoprotein-related protein 4 (LRP4) (3), antibodies to nicotinic acetylcholine receptor (nAChR) are the most common; they are detected in 85–90% of patients with generalized MG and in 50% of those with ocular MG (4). Prior research has revealed that nAChRs also exists on outer hair cells (OHCs) of the ears (5). OHCs receive efferent projections from the medial olivocochlear (MOC) bundle, whose endings are anatomically similar to those at the neuromuscular junction (6). Synapses between the neural endings of the MOC bundle and the receptors present on the basolateral membrane of OHCs are mainly cholinergic (7). Acetylcholine (ACh) increases the amplitude of electromotility in OHCs and is considered to be the main neurotransmitter of the efferent auditory system (5–7).

Otoacoustic emissions (OAEs) are low-level sounds originating in the cochlea and produced by active micromechanisms of the OHCs in the organ of Corti, thus constituting part of the normative hearing process (8). OAEs can detect fine changes in the OHCs that are undetectable by other audiological methods (5). Several studies have found evidence for a reduction of both distortion product OAEs (DPOAEs) and transiently evoked OAEs (TEOAEs) in MG patients relative to normal controls (6, 9–11). Interestingly, OAEs of such patients can be improved by treatment with reversible acetylcholinesterase (AChE) inhibitors (6, 9, 11).

Despite the evidence for an association between impaired OAE and MG, no prior study has—to the best of our knowledge—explored the association of OAE abnormalities with clinical features of MG patients. The present study therefore aimed to discern whether OAEs are correlated with clinical characteristics as well as electrophysiological and serological data to evaluate clinical implications of OAEs in MG.

## Materials and Methods

### Subjects

We recruited 20 patients with MG and conducted the study using 15 patients (nine women) with normal hearing, which was assessed by pure tone audiometry. Examinations were performed on both ears of each participant (30 ears). The mean age and duration of illness of the patients were 35.5 ± 10.5 years (range 18–49) and 16.2 ± 3.1 months (range 12–24), respectively. All patients were treated with individualized doses of immunotherapy with steroid and/or azathioprine and an AChE inhibitor, pyridostigmine bromide (mestinon). MG was diagnosed based on the presence of a typical history and at least one positive supplementary test—for example, the presence of serum anti-AChR antibody or a decremental pattern on repetitive nerve stimulation (RNS). Patients were recruited from two university-affiliated hospitals in Korea (Dongguk University Ilsan Hospital and Seoul National University Bundang Hospital) from April 2014 to February 2015. The control group was composed of 20 ears of 10 healthy age- and sex-matched participants (mean age 33.3 ± 2.5 years, range 29–38) without any neuromuscular or otological disorders.

Informed consent was obtained from all subjects. All experiments were approved by the institutional review boards of two university-affiliated hospitals and were performed in accordance with the Declaration of Helsinki.

### Audiological Evaluation

Basic audiological evaluations, including pure-tone audiometry, speech audiometry, tympanometry, and acoustic reflex, were performed. Pure tone air and bone conduction audiometry was conducted using a Madsen Aurial Plus audiometer (Madsen Electronics, Copenhagen, Denmark); we thus determined air conduction hearing thresholds for octave frequencies between 250 and 8,000 Hz and bone conduction thresholds for frequencies between 250 and 4,000 Hz. Tympanometry and acoustic reflex tests were elicited contralaterally using an Interacoustic Az7 (Interacoustics, Middelfart, Denmark). All subjects exhibited normal hearing thresholds for all frequencies tested, as well as normal tympanograms and acoustic reflexes.

### Otoacoustic Emission Testing

Examinations were performed after a 12-h drug-free period because the mean half-life of the plasma level after oral dosing of reversible AChE inhibitor (pyridostigmin) is 200 min (12). DPOAEs and TEOAEs were recorded and analyzed in both ears for all subjects in an acoustically isolated room using an ILO-v6 clinical OAE system (Otodynamics Ltd., London, UK). To assess the DPOAE, we simultaneously delivered two 70 dB SPL equilevel primary signals (f1 and f2) through a catheter inserted in the external auditory canal. The f1:f2 ratio was automatically set to 1.22. Levels of the 2fl-f2 DPOAE were depicted as a function of frequency: nine frequencies from 635 to 4,052 Hz constituted a DPOAE-gram. Data were evaluated on the assumption that the mean geometric frequency of the stimuli corresponded to the specific cochlear region considered responsible for the distortion products. Othodynamics DPOAE software sets the significance level for the DPOAE at 2 standard deviations (SDs) above the mean noise level (95% confidence). For TEOAE, 80 μs non-linear, unfiltered rarefaction clicks were provided by default protocol. Stimulus waveform intensity was expressed as decibel peak sound pressure level measured in the external ear canal at 80 dB SPL and a repetition rate of 16 clicks/s. TEOAE were analyzed during the 20 ms following stimulation onset, averaging 260 responses in each recording session. The emission response (in dB SPL) from each ear was used as a variable.

### Statistical Analysis

Wilcoxon signed-rank tests showed no significant difference in OAE levels between the right and left ears. Therefore, the OAE levels were averaged across the right and left ears to avoid increasing type I error according to the recommendation of Coren and Hakstian (13, 14).

Quantitative data are presented as arithmetic means, with error bars representing standard error of mean (SEM), or as median and interquartile ranges (25 to 75th percentiles). The use of either representation method depended on the distribution of the data. Mann-Whitney *U*-test or Kruskal-Wallis test was employed for statistical comparison among different groups. Correlations were assessed with Spearman's rank correlation and partial correlation analysis (adjusted for age) using IBM Statistical Package for the Social Sciences (software version 22) and GraphPad Prism (version 7).

## Results

**Figure 1:**
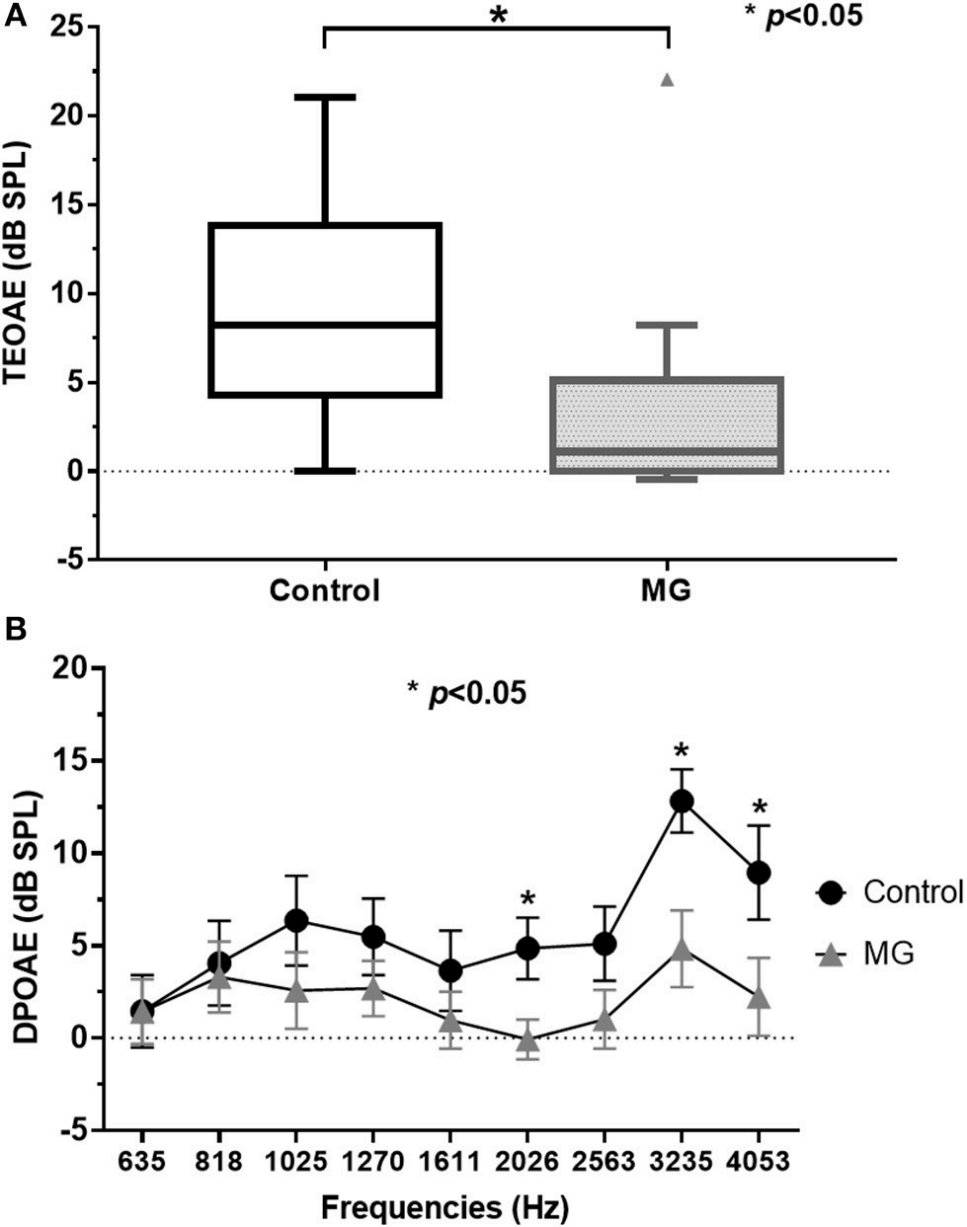
TEOAE and DPOAE amplitude values for controls and myasthenia gravis patients; **(A)** TEOAE amplitudes were significantly lower in MG patients (*n* = 15) relative to controls (*n* = 10, *p* < 0.05). **(B)** DPOAE amplitudes in MG were also lower compared to controls. These differences were significantly evident in higher frequencies (between 2,026 and 4,053 Hz) (*p* < 0.05). TEOAE, transiently evoked otoacoustic emissions; DPOAE, distortion product otoacoustic emissions; MG, myasthenia gravis.

Table 1 summarizes the clinical and diagnostic characteristics of the study population. TEOAE amplitudes across the 30 ears of the MG patients were significantly lower relative to those of healthy controls (*p* < 0.05) (Figure 1A; Supplemental Table 1). The means of the TEOAE amplitude of MG patients and controls were 3.41 ± 5.86 dB SPL and 8.69 ± 6.37 dB, respectively. The amplitude values of the DPOAE in MG also featured a decrease relative to controls; this observation was significantly evident at higher frequencies (between 2,026 and 4,053 Hz, *p* < 0.05) (Figure 1B; Supplemental Table 2).

**Table 1:** Baseline characteristics of the study population.

	**Myasthenia gravis group**	**Control group**
Number of subjects	15	10
Number of ears	30	20
Ages, years (*SD*, range)^*^	35.5 (10.5, 18–49)	33.3 (2.5, 29–38)
Disease duration, months (*SD*, range)	16.2 (3.1, 12–24)	
Positive AChR antibody (%)	24 (85.7)	
Mean AChR antibody titer, nM (*SD*, range)	5.18 (4.7, 0.01–13.30)	
Positive RNS test (%)	24 (80)	
Thymoma (%)	14 (46.7)	
Mechanical ventilator use (%)	8 (26.7)	
Pure ocular symptoms (%)	10 (33)	

* *p = 0.279. SD, standard deviation; AChR, acetylcholine receptor; RNS, repetitive nerve stimulation*.

**Figure 2:**
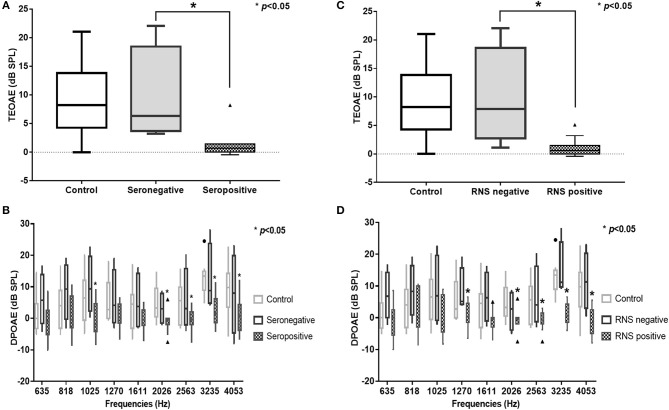
TEOAE and DPOAE amplitudes were significantly lower in the AChR antibody-positive (*n* = 12) **(A,B)** and the RNS-positive groups (*n* = 12) **(C,D)**, respectively (*p* < 0.05). TEOAE, transiently evoked otoacoustic emissions; DPOAE, distortion product otoacoustic emissions; AChR, acetylcholine receptor; RNS, repetitive nerve stimulation.

**Figure 3:**
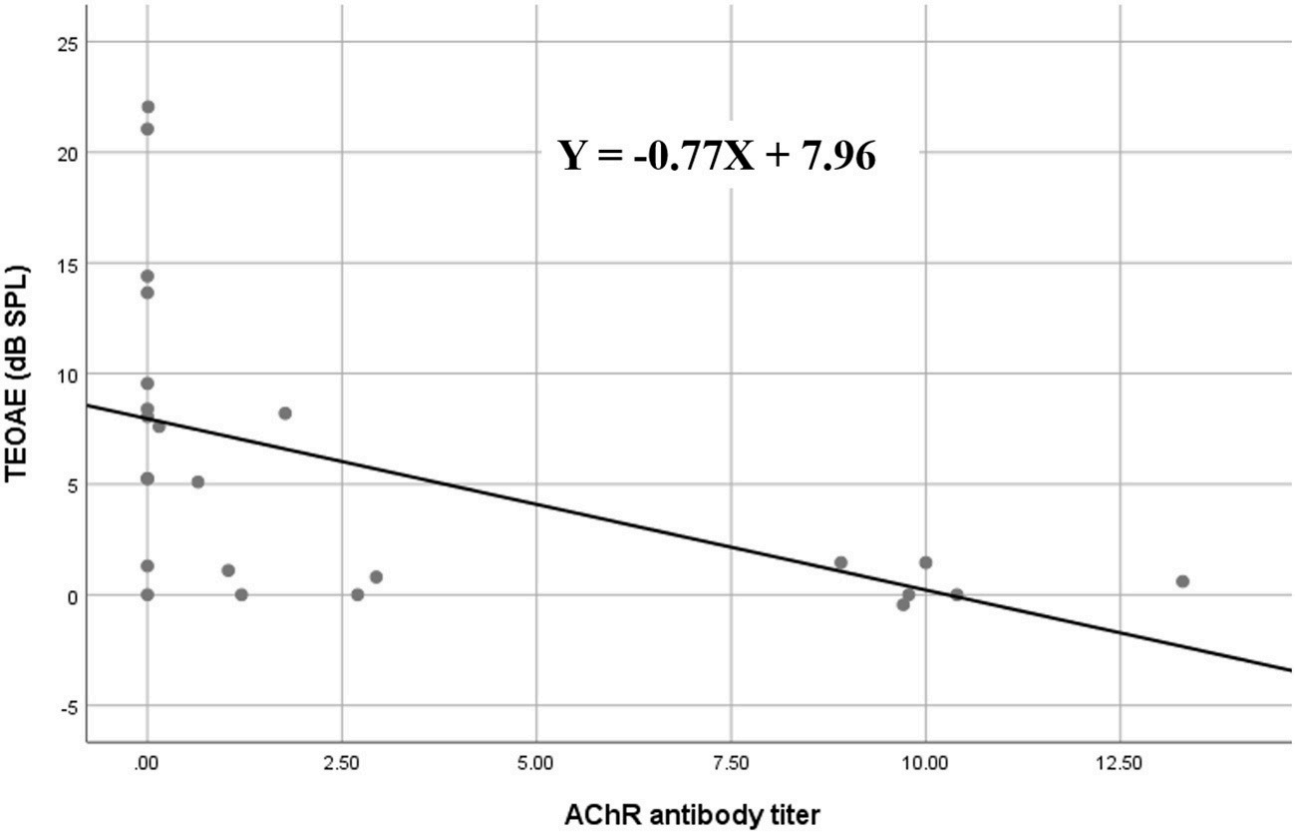
Spearman's rank correlation between AChR antibody titers and TEOAE amplitude values in myasthenia gravis; the amplitudes of TEOAEs significantly correlated with antibody titers. AChR, acetylcholine receptor; TEOAE, transiently evoked otoacoustic emissions.

In the subgroup analysis, DPOAE and TEOAE amplitudes were significantly lower than those in the positive anti-AChR antibody (*n* = 24, *p* < 0.05) and positive-RNS groups (*n* = 24, *p* < 0.05), respectively (Figures 2A–D; Supplemental Tables 1, 2). TEOAE amplitudes were consistently correlated with antibody titers (*r* = −0.631, *p* = 0.001), while DPOAE amplitudes were significantly correlated with antibody titers at middle and higher frequencies (1,025 Hz, *r* = −0.417, *p* = 0.042; 2,026 Hz, *r* = −0.520, *p* = 0.009; 3,235 Hz, *r* = −0.797, *p* < 0.001; 4,053 Hz, *r* = −0.501, *p* = 0.013) (Figure 3; Supplemental Table 3; Supplemental Figure 1). No significant difference in the OAEs was found between thymomatous and non-thymomatous MG, nor between purely ocular and generalized MG. Neither did we find a correlation between OAE and disease duration.

## Discussion

MG is the most common autoimmune disorder affecting the neuromuscular junction, causing intermittent muscle weakness that often worsens throughout the day or after prolonged activity (1). Despite relatively stable incidence rates, epidemiological studies have indicated an upward trend in the prevalence of MG (15). The disorder initially affects extrinsic ocular muscles in about two-thirds of patients (16), ~50% of whom develop generalized MG within 2 years (16). The most commonly used immunological test to diagnose MG is the measurement of serum concentrations of the AChR antibody (17). Although this test is relatively sensitive and specific for MG, it is useless for about 15% of patients with generalized MG and up to 50% of patients with ocular MG (17). Moreover, serum concentrations of AChR antibodies do not correlate with the severity of disease (16, 18). A means by which to diagnose MG reliably is therefore necessary.

As evidence of the association between auditory function and MG is accumulating, the efficacy of auditory tests as ancillary diagnostic techniques for MG is becoming more plausible (5). OAEs have revealed subclinical hearing alterations in patients with MG and could therefore be used as a tool for monitoring OHC function in individuals with MG (6, 9–11). We observed that TEOAEs featured consistently lower amplitudes in patients with MG relative to controls (Figure 1A), while the DPOAE amplitudes were significantly lower in patients with MG than in controls for middle and high frequencies (between 2,026 and 4,053 Hz) (Figure 1B). These findings are consistent with those of previous studies (6, 9–11), confirming that OAEs may be used to detect early subclinical auditory dysfunction in MG patients. Furthermore, the DPOAE and TEOAE amplitudes in AChR antibody-positive or RNS-positive groups significantly decreased compared to AChR antibody-negative and RNS-negative group, respectively, as well as control groups (Figures 2A–D). In particular, the amplitudes of both DPOAEs and TEOAEs significantly correlated with antibody titers (Figure 3; Supplemental Table 3; Supplemental Figure 1). These results indicate that AChR antibodies of MG patients might bind to AChRs on OHCs (10, 19) and that the diminishment of OHC function might occur according to the amount of antibody and the degree of the Ach-receptor dysfunction in the muscle membrane. This study therefore found strong evidence for an association between OAEs and electrophysiological and serological characteristics of MG.

The aforementioned auditory impairment might be linked to the disruption of the cholinergic synaptic transmission between MOC efferent fibers and OHCs, resembling that which occurs at the neuromuscular junction. AChR is classified into two major subtypes according to their pharmacological and molecular properties: muscarinic AChRs and nAChRs (20), the latter of which consists of subunits that vary according to the crucial physiologic roles the receptor plays throughout the central and peripheral nervous system (21). In an early immunohistochemial experiment, Plinkert et al. localized postsynaptic transmembraneous nAChRs presenting at the base of OHCs in the cochlea from fetal guinea pigs using nAChR antibodies (19, 22). For several decades after its initial discovery in the auditory system, the role of nAChR in normal hearing has been gradually elucidated. In healthy subjects, ACh has been found to enhance the electromotility of OHCs binding to nAChRs. In patients with MG, a reduced electromotility of OHCs was observed, but was reversible by ACh (5). It is now accepted that synaptic transmission between efferent olivocochlear fibers and OHCs of the cochlea is mediated by unique α9α10 nAChRs (20, 23). Intriguingly, the α1 nicotinic receptor subunit, which is known to mediate cholinergic inputs in muscle and to be main target of autoantibodies in MG (24), has also been documented to mediate the aforementioned synaptic transmission (25, 26). Furthermore, α-bungarotoxin which binds the same site of nAChR antibody of patients with MG was reported to bind the nAChRs on OHCs in addition to those on muscle membrane (19). These mechanisms may account for the association between OAE abnormalities in patients with MG and the nAChR dysfunction of OHCs that diminishes OHC electromotility.

As MG is a rare disease, the present study is subject to the limitation of small sample sizes; however, the mean differences in OAEs observed in the present study are relatively large. Despite no statistically significant difference in mean age, it is also worth noting that there was a relatively wide age range of the study population relative to that of the controls. Our findings therefore warrant larger prospective studies to further characterize the correlation between OAEs and the clinical features of MG. In addition, it would be better to confirm the binding of antibody from MG patients to OHCs directly to clarify the pathological correlation. Such research would help to discern the potential of OAE assessment as a supplementary diagnostic and monitoring tool for MG.

## Conclusions

This study provides support for the role of ACh in the efferent function of OHC, as well as the impaired function of nAChRs on OHCs of patients with MG. Furthermore, to the best of our knowledge, this report is the first to document more reduced OAEs in RNS-positive and the AChR antibody-positive groups. In particular, our analysis of the correlation between OAE alterations in patients with MG and their serum levels of anti-nAChR antibodies indicates that OHC function of patients with MG can be impaired by AChR antibodies. Our findings further suggest that OAE measurements can increase the diagnostic certainty of MG and help to monitor its severity.

## Author Contributions

JC, N-HK, and KSP contributed at all stages of manuscript preparation. All authors were involved in data recording and discussed the results. KSP critically appraised and approved the final manuscript.

### Conflict of Interest Statement

The authors declare that the research was conducted in the absence of any commercial or financial relationships that could be construed as a potential conflict of interest.

## Abbreviations

ACh: acetylcholine
AChR: acetylcholine receptor
AChE inhibitors: acetylcholinesterase inhibitors
DPOAEs: distortion product otoacoustic emissions
LRP4: lipoprotein-related protein 4
MOC bundle: medial olivocochlear bundle
MuSK: muscle-specific kinase
MG: Myasthenia gravis
nAChRs: nicotinic AChR acetylcholine receptors
OAEs: otoacoustic emissions
OHCs: outer hair cells
RNS: repetitive nerve stimulation
SD: standard deviation
SEM: standard error of mean
TEOAEs: transiently evoked otoacoustic emissions.
