# Supplementary material for: Abnormalities of Otoacoustic Emissions in Myasthenia Gravis: Association With Serological and Electrophysiological Features
Source: Front Neurol. 2018 Dec 20;9:1124. doi: 10.3389/fneur.2018.01124 (PMC6306561; doi:10.3389/fneur.2018.01124)
Supplement: Supplementary file 1 [file Data_Sheet_1.docx]

**Table 1. TEOAE amplitude values for controls and myasthenia gravis patients**

| **Group** | **TEOAE (dB SPL)*** | ***P†*** |
| --- | --- | --- |
| Normal controls (n = 10) | 8.69±6.368 | - |
| MG patients (n = 15) | 3.407±5.855 | **0.0436** |
| Seronegative MG (n = 3) | 9.488±8.567 | 0.8502 |
| Seropositive MG (n = 12) | 1.315±2.507 | **0.0139** |
| RNS negative MG (n = 3) | 9.738±8.815 | 0.8062 |
| RNS positive MG (n = 12) | 1.105±1.68 | **0.0012** |

*Summarized as mean±SD; †Calculated using a Mann-Whitney U test. Abbreviations: TEOAE, transiently evoked otoacoustic emissions; MG, myasthenia gravis; RNS, repetitive nerve stimulation.

**Table 2. DPOAE amplitude values for controls and myasthenia gravis patients**

|  | **635 Hz** | **818 Hz** | **1025 Hz** | **1270 Hz** | **1611 Hz** | **2026 Hz** | **2563 Hz** | **3235 Hz** | **4053 Hz** |
| --- | --- | --- | --- | --- | --- | --- | --- | --- | --- |
| Controls (n = 10) | 1.45±6.189 | 4.050±7.259 | 6.350±7.664 | 5.475±6.541 | 3.650±6.876 | 4.850±5.250 | 5.100±6.352 | 12.825±5.414 | 8.950±8.040 |
| MG patients (n = 15) | 1.433±6.800 | 3.300±7.428 | 2.567±8.009 | 2.683±5.811 | 0.967±5.969 | -0.067±4.157 | 1.017±6.120 | 4.833±8.040 | 2.233±8.159 |
| ***P†*** | 0.99 | 0.80 | 0.25 | 0.27 | 0.31 | **0.01** | 0.12 | **0.01** | **0.04** |
| Seronegative MG (n = 3) | 6.000±8.377 | 8.625±9.068 | 10.438±9.161 | 6.063±9.270 | 5.125±9.003 | 3.125±4.939 | 5.625±10.117 | 12.438±10.748 | 7.750±12.823 |
| ***P†*** | 0.28 | 0.33 | 0.40 | 0.89 | 0.74 | 0.58 | 0.90 | 0.92 | 0.83 |
| Seropositive MG (n = 12) | -0.542±5.529 | 1.000±5.943 | -0.229±5.300 | 1.083±3.940 | -0.625±3.838 | -1.125±3.227 | -0.354±3.217 | 2.438±4.871 | 0.667±5.206 |
| ***P†*** | 0.43 | 0.29 | **0.02** | 0.06 | 0.08 | **0.003** | **0.01** | **0.0001** | **0.008** |
| RNS negative MG (n = 3) | 7.000±7.583 | 8.500±8.377 | 8.563±10.732 | 8.313±7.151 | 6.500±7.885 | 2.250±6.144 | 6.313±9.683 | 14.875±8.795 | 11.500±9.211 |
| ***P†*** | 0.17 | 0.33 | 0.66 | 0.48 | 0.51 | 0.43 | 0.78 | 0.59 | 0.61 |
| RNS positive MG (n = 12) | -0.591±5.531 | 1.409±6.437 | 0.386±5.970 | 0.636±3.826 | -1.045±3.811 | -0.909±3.153 | -0.909±3.003 | 1.182±3.506 | -1.136±4.572 |
| ***P†*** | 0.43 | 0.38 | 0.06 | **0.04** | 0.06 | **0.00613** | **0.01104** | **0.00001** | **0.0020** |

*Summarized as mean±SD; †Calculated using a Mann-Whitney U test. Abbreviations: DPOAE, distortion product otoacoustic emissions; MG, myasthenia gravis; RNS, repetitive nerve stimulation.

**Table 3. Spearman’s rank correlations between AChR antibody titers and OAE amplitude values in myasthenia gravis**

|  | **Y = b + aX** | **r** | ***P*** |
| --- | --- | --- | --- |
| **TEOAE** | Y = 7.96 – 0.77X | -0.631 | **0.001** |
| **DPOAE** |  | | |
| **635 Hz** | Y = 2.33 – 0.22X | -0.052 | 0.810 |
| **818 Hz** | Y= 4.87 – 0.33X | -0.080 | 0.711 |
| **1025 Hz** | Y = 6.3 – 0.68X | -0.417 | **0.042** |
| **1270 Hz** | Y = 5.93 – 0.61X | -0.303 | 0.150 |
| **1611 Hz** | Y = 3.68 – 0.49X | -0.307 | 0.145 |
| **2026 Hz** | Y = 3.64 – 0.55X | -0.520 | **0.009** |
| **2563 Hz** | Y = 4.63 – 0.66X | -0.397 | 0.055 |
| **3235 Hz** | Y = 12.08 – 1.32X | -0.797 | **<0.001** |
| **4053 Hz** | Y = 7.63 – 0.9X | -0.501 | **0.013** |

Abbreviations: AChR, acetylcholine receptor; OAE, otoacoustic emissions; TEOAE, transiently evoked otoacoustic emissions; DPOAE, distortion product otoacoustic emissions.

**Figure 1. Spearman’s correlations between AChR antibody titers and DPOAE amplitude values in myasthenia gravis**


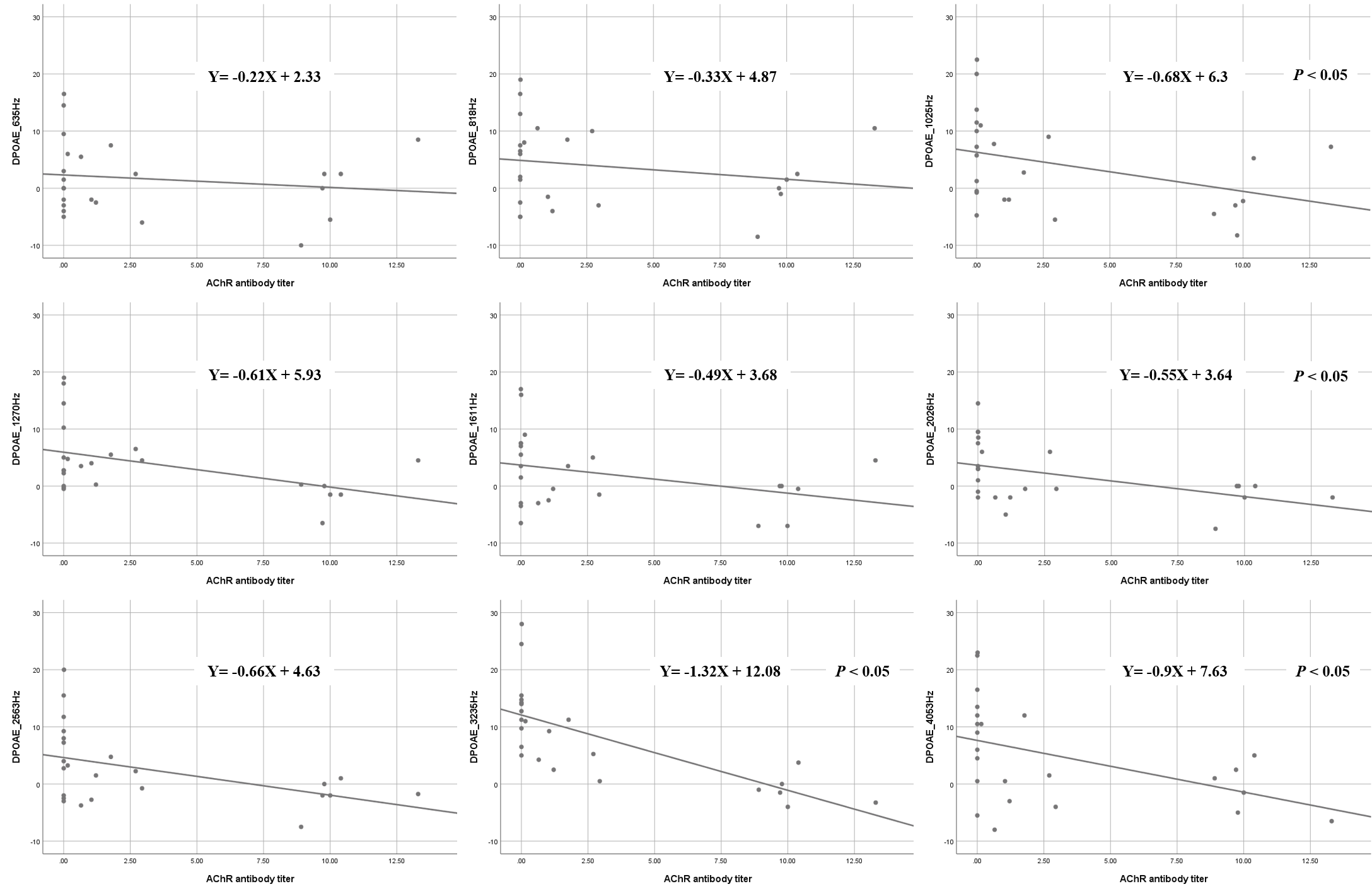


Abbreviations: AChR, acetylcholine receptor; DPOAE, distortion product otoacoustic emissions.
